# Adaptive Governance: Empowering Data Sharing for Public Health Impact - Insights from North Carolina Department of Health and Human Services

**DOI:** 10.23889/ijpds.v9i5.2569

**Published:** 2024-09-18

**Authors:** Amy Hawn Nelson, Paul Hogle, Sharon Zanti, Scott Proescholdbell, Jessie Tenenbaum

**Affiliations:** 1 University of Pennsylvania; 2 North Carolina Department of Health & Human Services; 3 Duke University

## Objective and Approach

At the 2022 IPDLN Conference, we presented work led by the North Carolina Department of Health and Human Services (NCDHSS), in partnership with university-based researchers, to build legal and ethical processes for routine data sharing. This session provides significant updates on the methods, results, and implications of these efforts–namely, that NCDHHS implemented an enterprise-wide data governance process and a legal framework that has enabled timely, impactful use of cross-sector data. We relied on Participatory Action Research and Deliberative Dialogue methods to engage a diverse range of partners in a data landscape overview and the co-creation of new data sharing processes that better enables the enterprise to adapt to a changing world. 

## Results

Four key actions were taken as a result of the participatory research process: NCDHHS developed a data strategy, created a data sharing guidebook, staffed their Data Office, and implemented a new legal framework. In addition to describing how these actions support data use across a large US state health and human services agency, we provide three use cases demonstrating the impact of this work.

## Conclusions

Establishing routine data sharing presents legal, technical, and cultural challenges, particularly in large  agencies. Through a collaborative, participatory approach, the NCDHHS successfully established enterprise-wide data governance and a legal framework to support data-driven policymaking and, ultimately, improve health outcomes for residents.

## Implications

This research presents a successful, actionable, and replicable framework for developing and implementing processes to support intradepartmental data access, linkage, and use.

